# Electrophysiological Effects of SKF83959 on Hippocampal CA1 Pyramidal Neurons: Potential Mechanisms for the Drug's Neuroprotective Effects

**DOI:** 10.1371/journal.pone.0013118

**Published:** 2010-10-01

**Authors:** Hong-Yuan Chu, Qinhua Gu, Guo-Zhang Jin, Guo-Yuan Hu, Xuechu Zhen

**Affiliations:** State Key Laboratory of Drug Research and Department of Pharmacology II, Shanghai Institute of Materia Medica, Chinese Academy of Sciences, Shanghai, China; University of Texas Health Science Center, United States of America

## Abstract

Although the potent anti-parkinsonian action of the atypical D_1_-like receptor agonist SKF83959 has been attributed to the selective activation of phosphoinositol(PI)-linked D_1_ receptor, whereas the mechanism underlying its potent neuroprotective effect is not fully understood. In the present study, the actions of SKF83959 on neuronal membrane potential and neuronal excitability were investigated in CA1 pyramidal neurons of rat hippocampal slices. SKF83959 (10–100 µM) caused a concentration-dependent depolarization, associated with a reduction of input resistance in CA1 pyramidal neurons. The depolarization was blocked neither by antagonists for D_1_, D_2_, 5-HT_2A/2C_ receptors and α_1_-adrenoceptor, nor by intracellular dialysis of GDP-β-S. However, the specific HCN channel blocker ZD7288 (10 µM) antagonized both the depolarization and reduction of input resistance caused by SKF83959. In voltage-clamp experiments, SKF83959 (10–100 µM) caused a concentration-dependent increase of *Ih* current in CA1 pyramidal neurons, which was independent of D_1_ receptor activation. Moreover, SKF83959 (50 µM) caused a 6 mV positive shift in the activation curve of *Ih* and significantly accelerated the activation of *Ih* current. In addition, SKF83959 also reduced the neuronal excitability of CA1 pyramidal neurons, which was manifested by the decrease in the number and amplitude of action potentials evoked by depolarizing currents, and by the increase of firing threshold and rhoebase current. The above results suggest that SKF83959 increased *Ih* current through a D_1_ receptor-independent mechanism, which led to the depolarization of hippocampal CA1 pyramidal neurons. These findings provide a novel mechanism for the drug's neuroprotective effects, which may contributes to its therapeutic benefits in Parkinson's disease.

## Introduction

SKF83959 (3-methyl-6-chloro-7,8-hydroxy-1-[3-methylphenyl]-2,3,4,5-tetrahydro-1H-3- benzazepine) is a selective agonist for the putative phosphatidylinositol (PI)-linked D_1_-like receptor [1]–[3]. It has been demonstrated that SKF83959 produces no cAMP formation in brain tissues but induces PI-hydrolysis via activation of the PI-linked D_1_-like receptor/Gq protein/PLCβ signaling pathway[1]–[5]. In the primate and rodent models of Parkinson's disease, chronic or sub-chronic administration of SKF83959 was found to produce potent therapeutic effects [6]–[8]. Moreover, chronic administration of this drug was found to attenuate the L-DOPA-induced dyskinesia (LID) in 6-OH-DOPA-lesioned rat models [6], [9], [10]. Although the anti-parkinsonian action of SKF83959 has been attributed to activation of PI-linked D_1_-like receptor [1], [8], [9], [11], the exact mechanisms underlying the action remain unclear.

Our previous work demonstrated that SKF83959 exerted a potent neuroprotective action in rat pheochromocytoma cells (PC12 cells) treated with H_2_O_2_ [12]. This action, however, was only partially attributed to inhibition of glycogen synthase kinase-3β (GSK3β) by SKF83959 via activation of D_1_-like receptor. Therefore, other mechanisms independent of D_1_-like receptor may be involved in the neuroprotection by SKF83959. Accumulating evidence shows that enhanced delayed rectifier K^+^ channel induces neuronal death, while blocking K^+^ outflow through the K^+^ channel promotes the survival of neurons [13]–[15]. We have shown recently that SKF83959 is a potent blocker of the delayed rectifier K^+^ channels in rat hippocampal pyramidal neurons [16], which may contribute to the non-receptor mechanisms of the neuroprotection of the drug. The membrane properties and excitability play critical roles in physiological and pathological activity of brain neurons. It has been demonstrated that an increased neuronal excitability in the pathological state of 6-OH-DOPA-lesioned rat model for Parkinson's disease [17]–[19]. Alongside our previous study [16], the present study was designed to further explore the effects of SKF83959 on the neuronal membrane properties and excitability in rat hippocampal pyramidal neurons. The present results demonstrate that SKF83959 not only induces membrane depolarization of CA1 pyramidal neurons via an enhancement of *Ih* current in a DA-receptor-independent manner, but also reduces the neuronal excitability. These findings may provide a novel mechanism for the drug's neuroprotective effects and its anti-Parkinsonian efficacy.

## Results

### Effects of SKF83959 on passive membrane properties of hippocampal CA1 pyramidal neurons

**Figure 1:**
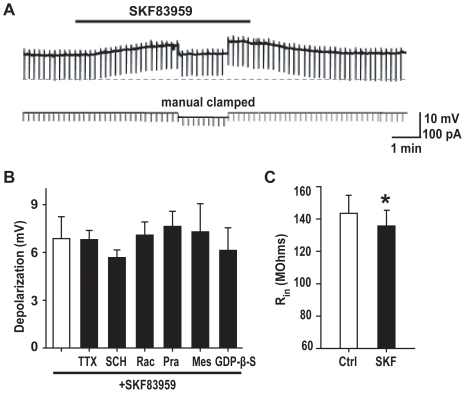
SKF83959 induced depolarizing response in CA1 pyramidal neuron in hippocampal slices. A. Resting membrane potential recorded in a representative neuron. The upper trace shows the membrane potential of the neuron. The resting potential was −60 mV, whereas the input resistance change was monitored in the upper trace through injecting hyperpolarizing current pulses (400 ms, −50 pA, lower trace) every 10 sec (the downward deflections). The black bar denotes the perfusion with SKF83959 (50 µM). To exclude the change of input resistance caused indirectly by depolarizing response, the membrane potential during SKF83959 application was manually clamped to the pre-drug level. Bicuculline (5 µM) was added in ASCF to suppress the spontaneous IPSPs. B. Bar graphs showing the maximal depolarization caused by SKF83959 (50 µM) in the presence of TTX (0.5 µM, n = 5), SCH (D1 receptor antagonist SCH23390, 10 µM, n = 5), Rac (D2 receptor antagonist raclopride, 10 µM, n = 5), Mes (5-HT2A/2C receptor antagonist mesulergine, 10 µM, n = 5), Pra (Alpha1-adrenoceptor antagonist prazosin, 10 µM, n = 5), or following intracellular dialysis of GDP-β-S (0.5 mM, n = 6). C. Bar graphs showing the input resistance in the control (Ctrl) and during perfusion with SKF83959 (SKF, 50 µM, n = 10, *P<0.05).

Bath application of SKF83959 caused a reversible depolarizing response of CA1 pyramidal neurons in rat hippocampal slices (Fig. 1A). The maximal responses caused by SKF83959 at the concentrations of 10, 50 and 100 µM were 2.5±0.4 mV (n = 5), 6.9±1.4 mV (n = 6) and 9.7±1.0 mV (n = 7), respectively. The depolarization never led to spontaneous firing of the recorded neuron even when the resting membrane potential was set to a level close to the threshold of action potential firing by injecting steady depolarizing current. The depolarization caused by SKF83959 (50 µM) persisted, when TTX (0.5 µM) was included in the perfusion medium (6.8±0.7 mV, n = 5, unpaired *t* test, *P*>0.05 *vs.* SKF83959 alone, Fig.1B). Depolarizing responses were also observed in acutely dissociated CA1 pyramidal neurons (PND5–6). Superfusion of SKF83959 (50 µM) caused a depolarization of 4.9±0.5 mV (n = 6) in dissociated single CA1 pyramidal neurons. The results suggest that the depolarization effect of SKF83959 was independent of synaptic connections.

SKF83959 is an atypical agonist of D_1_-like receptor, and also exhibits moderate or weak affinity to D_2_ receptor, α_1_-adrenoceptor and 5-HT_2A/2C_ receptor [5]. We tested whether any of those receptors are responsible for the effect of SKF83959. As shown in Fig. 1B, the maximal depolarization caused by SKF83959 (50 µM) in the presence of D_1_ receptor antagonist SCH23390 (10 µM) was not significantly altered (SCH23390+SKF83959: 5.7±0.5 mV, n = 8; SKF83959 alone: 6.9±1.4 mV, n = 6, unpaired *t* test, *P*>0.05). Similar results were obtained in the presence of D_2_ receptor antagonist raclopride (10 µM, n = 5), 5-HT_2A/2C_ receptor antagonist mesulergine (10 µM, n = 5) or α_1_-adrenoceptor antagonist prazosin (10 µM, n = 5) (Fig. 1B). Furthermore, intracellular dialysis of 0.5 mM GDP-β-S, a hydrolysis-resistant GDP analog which blocked G-protein activation, was also found to produce no significant effect on SKF83959-induced depolarizing response (GDP-β-S +SKF83959: 6.1±1.4 mV, n = 6; SKF83959 alone: 6.9±1.4 mV, n = 6, unpaired *t* test, *P*>0.05). These results further support the observation that SKF83959-induced depolarization was not mediated through the activation of D_1_ receptor and other related G-protein coupled receptors.

In a representative experiment shown in Fig. 1A, the membrane potential was manually clamped to the control level during SKF83959 application to monitor the change of input resistance of the recorded neurons. The pooled data from the 10 neurons shows that SKF83959 (50 µM) caused a small but statistically significant reduction of the input resistance (from 143.5±11.2 MΩ to 135.7±9.5 MΩ, paired *t* test, *P*<0.05, Fig. 1C). The results suggest that SKF83959-induced membrane depolarization was accompanied by a reduction of the input resistance.

### Effects of SKF83959 on subthreshold responses of hippocampal CA1 pyramidal neurons

**Figure 2:**
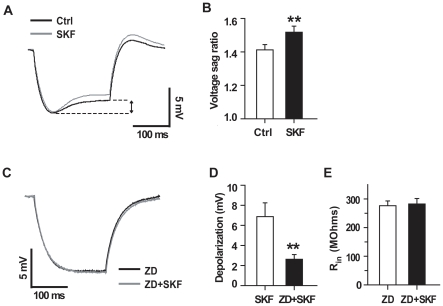
Effects of SKF83959 on subthreshold response of CA1 pyramidal neurons. A. Superimposed responses to prolonged hyperpolarizing current pulses (400 ms, −50 pA) recorded from a representative neuron before (black) and during (gray) the perfusion with SKF83959 (50 µM), showing the enhanced voltage sag (arrow). The resting potential of the neuron was −60 mV, and the membrane potential was manually clamped to compensate the SKF83959-induced depolarizing response. B. Bar graphs showing the voltage sag ratios in the presence and absence of SKF83959 (50 µM). The voltage sag ratio was quantified as the peak voltage deflection divided by the steady-state voltage deflection. C. The results were obtained from another neuron in the presence of ZD7288 (10 µM). The resting potential of the neuron was −66 mV. Note that ZD7288 completely abolished the voltage sag. D. Bar graphs showing the maximal depolarizing response caused by SKF83959 (50 µM) in the presence (n = 6) and absence (n = 8) of ZD7288 (10 µM). E. Bar graphs showing the input resistance of CA1 pyramidal neurons measured in the presence of ZD7288 (10 µM) or in the presence of ZD7288 (10 µM) and SKF83959 (50 µM). n = 8 for each group. ** P<0.01. Ctrl, Control; SKF, SKF83959; ZD, ZD7288.

The hyperpolarization-activated non-selective cation current (*Ih*), which is mediated by the hyperpolarization-activated, cyclic-neucleotide gated (HCN) channels, plays a crucial role in setting the resting membrane potential of neurons [20]. To explore the mechanism underlying SKF83959-induced depolarization, we examined the effect of SKF83959 on the voltage sag caused by prolonged hyperpolarizing current pulse, which is a hallmark of I*h* activation [21]. In the presence of SKF83959 (50 µM), injecting the hyperpolarizing current pulses produced more pronounced voltage sags (Fig. 2A). In a group of neurons tested, the voltage sag ratio was significantly increased from 1.4±0.03 to 1.5±0.03 (n = 10, paired *t* test, *P*<0.01, Fig. 2B).

In order to confirm the involvement of *Ih* in SKF83959-induced depolarization, we pretreated the slice with ZD7288, a specific HCN channel blocker, and found that ZD7288 (10 µM) completely abolished the voltage sag either in the absence or in the presence of SKF83959 (Fig. 2C). Furthermore, inhibition of *Ih* by ZD7288 (10 µM) significantly antagonized both the depolarizing response and input resistance reduction caused by SKF83959. As shown in Figs. 2D, 2E, the maximal depolarizing response caused by SKF83959 (50 µM) alone was 6.9±1.4 mV (n = 6), whereas SKF83959 induced depolarization response was significantly reduced to 2.6±0.5 mV (n = 8, unpaired *t* test, *P*<0.01) in the presence of ZD7288. Moreover, pretreatment with ZD7288 (10 µM) drastically increased the input resistance of the neurons tested to 276.3±16.5 MΩ (n = 8). However, subsequent perfusion with SKF83959 failed to change the neuronal input resistance in the presence of ZD7288 (n = 8, 282.4±19.2 MΩ, paired *t* test, *P*>0.1). The above results suggest that activation of *Ih* is responsible for SKF83959-induced depolarization.

### SKF83959 enhanced Ih current in hippocampal CA1 pyramidal neurons

**Figure 3:**
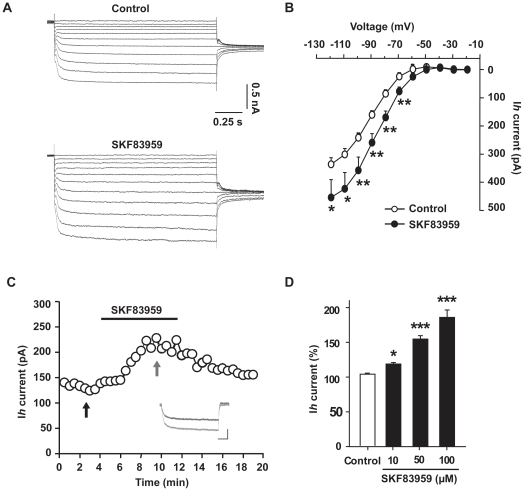
SKF83959 increased Ih current in hippocampal CA1 pyramidal neurons. A. Current family of Ih recorded from a representative neuron in the presence and absence of SKF83959 (50 µM). The neuron was hold at −20 mV and current traces were elicited with a series of 1.5-s hyperpolarizing voltage steps from −20 mV to −120 mV with increment of 10 mV followed by a voltage step to −80 mV to measure the tail currents. B. Averaged current/voltage (I/V) relationship of Ih plotted in the presence and absence of SKF83959 (50 µM). C. Plot of the amplitude of Ih against time in a representative neuron. The black bar denotes the perfusion with SKF83959 (50 µM). The neuron was hold at −45 mV, and Ih was elicited with 1.5-s hyperpolarizing voltage steps to −105 mV every 30 sec. The inset shows the superimposed current traces taken at the time indicated by the two arrows. Scale bars: 0.5 s, 250 pA. D. Bar graphs showing the maximal steady-state amplitude of Ih in the presence of different concentrations of SKF83959. *P<0.05, **P<0.01, ***P<0.001 vs. Control.

The above data indicated that SKF83959 enhances the activity of HCN channels. Consistently, perfusion with SKF83959 (50 µM) markedly increase the amplitude of *Ih* of CA1 pyramidal neurons in hippocampal slice at all the potentials tested (Fig. 3A). Plot of the averaged current/voltage (*I/V*) relationship of *Ih* in the presence or absence of SKF83959 reveals a down-shift of the *I/V* curve, and an enhancement of the maximal steady-state current amplitude (from −70 to −120 mV, Fig. 3B). The amplitude of *Ih* at −120 mV was increased from 336±23 pA to 454±63 pA (n = 6, paired *t* test, *P*<0.05). Moreover, SKF83959-induced enhancement of *Ih* was reversible (Fig. 3C) and in a dose-dependent manner (Fig. 3D). At the concentrations of 10, 50, and 100 µM, SKF83959 increased the amplitude of *Ih* by 18.9±2.0% (n = 5, ANOVA, *P*<0.05), 54.7±5.0% (n = 11, ANOVA, *P*<0.001) and 85.9±10.6% (n = 6, ANOVA, *P*<0.001), respectively.

**Figure 4:**
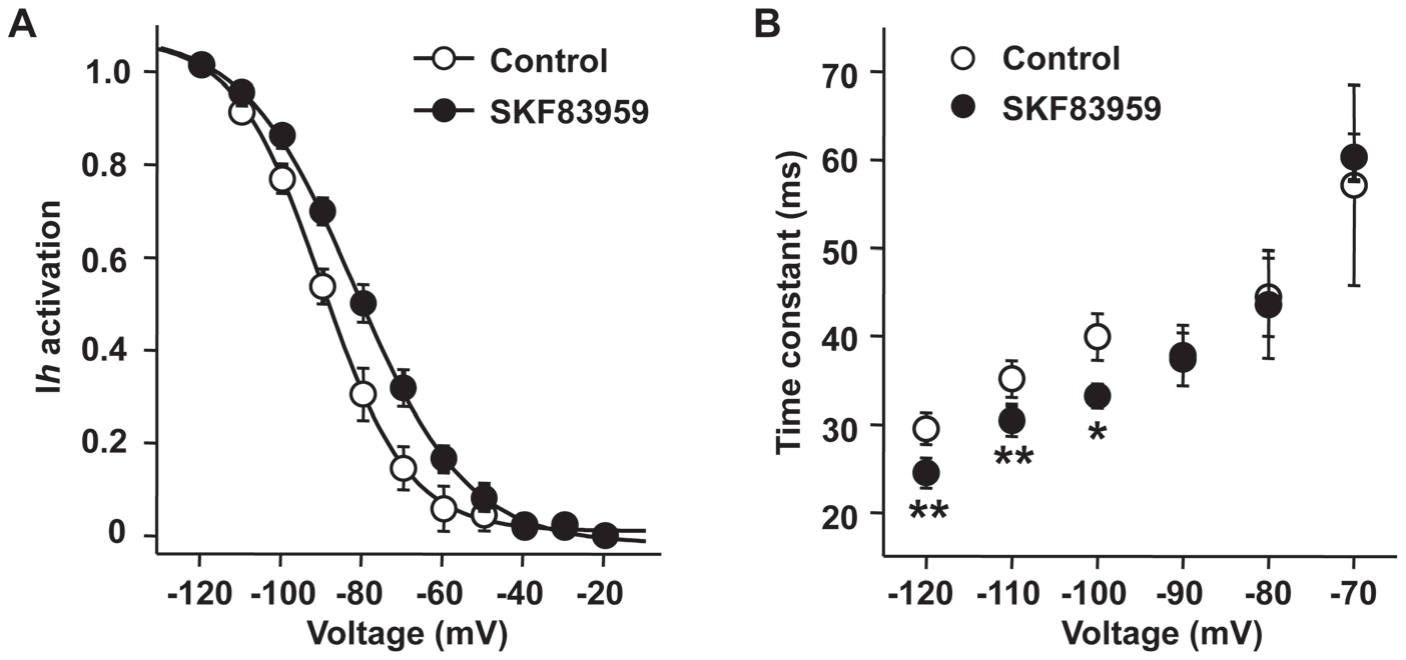
Effects of SKF83959 on the kinetic properties of Ih current. A. the activation curves of Ih plotted in control and in the presence of SKF83959 (50 µM). The neurons were held at −20 mV. Ih currents were elicited with 1.5-sec hyperpolarizing steps to various potentials followed by a voltage step to −80 mV to measure the tail currents. Normalized amplitude of the tail current was plotted as the function of the test potentials and fitted with the Boltzmann equation: I/Imax = 1/[1+exp(V-V1/2)/s], where I/Imax is the normalized amplitude of the tail current, V is the test potential, V1/2 is the half-activation potential, and s is the slope factor. B. Plot of the activation time constant (τf) of Ih against the test potentials. The trace of Ih current was fitted with bi-exponential functions. n = 6 for each symbol. *P<0.05, **P<0.01 vs. Control.

In order to elucidate the mechanisms for the drug-mediated enhancement of *Ih*, we first examined whether SKF83959 modulates the gating mechanisms of *Ih* channels. Perfusion with SKF83959 (50 µM) caused a right shift of the activation curve of *Ih* (Fig. 4A). In the control period, the half-activation potential for *Ih* (*V*_1/2_) was −90.8±1.8 mV (n = 6), and the slop factor was 11.5±1.0 (n = 6). In the presence of SKF83959, the value of *V*_1/2_ changed to −84.2±1.7 mV (n = 6, paired *t* test, *P*<0.001), and the slop factor to 14.1±1.1 (n = 6, paired *t* test, *P*<0.01). Furthermore, SKF83959 accelerated the activation of *Ih* in steps to large hyperpolarizing voltage steps (from −120 to −100 mV) (Fig. 4B). The *Ih* current traces could be fitted with bi-exponential functions with a relatively stable fast component, which accounted for the majority of *Ih*, followed by a variable slow component. The fast activation time constant (τ_f_) of *Ih* in steps to −120, −110 and −100 mV in the control period were 29.5±1.8 ms, 35.2±2.1 ms and 39.9±2.6 ms, respectively; whereas subsequent perfusion with SKF83959 (50 µM), the values of τ_f_ were significantly reduced to 24.5±1.7 ms (n = 6, paired *t* test, *P*<0.01), 30.4±1.7 ms (n = 6, paired *t* test, *P*<0.01) and 33.2±1.7 ms (n = 6, paired *t* test, *P*<0.05), respectively.

**Figure 5:**
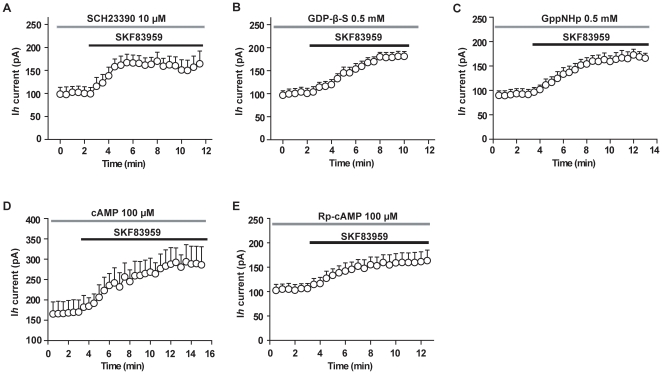
Increase of Ih by SKF83959 was independent of activation of D1-like receptors. The neurons were held at −45 mV, and Ih current was elicited with 1.5-s hyperpolarizing voltage steps to −105 mV every 30 sec. In each panel, the amplitude of Ih was plotted against time. The black bars denotes the perfusion with SKF83959 (50 µM), whereas the gray bar denotes the application of various agents: A. perfusion with D1-like receptor antagonist SCH23390 (10 µM), n = 11; B. Intracellular dialysis of GDP-beta-S (0.5 mM), n = 9; C. intracellular dialysis of GppNHp (0.5 mM), n = 6; D. intracellular dialysis of high concentrations of cAMP (100 µM), n = 6; E. intracellular dialysis of Rp-cAPM (100 µM), n = 6.

We then examined whether the enhancement of *Ih* by SKF83959 was mediated through activation of D_1_-like receptors. As shown in Fig. 5A, in the presence of D_1_-like receptor antagonist SCH23390 (10 µM), perfusion with SKF83959 (50 µM) increased the amplitude of *Ih* by 62.9±12.4% (n = 6, paired *t* test, *P*<0.05), which was close to that obtained in the absence of SCH23390 (54.7±5.0%, unpaired *t* test, n = 11). Intracellular dialysis of GDP-β-S did not prevent the enhancement of *Ih* by SKF83959 either (Figs. 5B). With GDP-β-S (0.5 mM) present in the recording pipettes, perfusion with SKF83959 (50 µM) increased the amplitude of *Ih* by 81.9±10.1% (n = 9, paired *t* test, *P*<0.001). Similar result was obtained with intracellular dialysis of GppNHp, a hydrolysis-resistant GTP analog, which uncoupled G-protein (Figs. 5C). With GppNHp (0.5 mM) present in the recording pipettes, perfusion with SKF83959 (50 µM) increased the amplitude of *Ih* by 79.3±10.8% (n = 6, paired *t* test, *P*<0.001).

It has been shown that the activation of *Ih* is facilitated by cAMP in a direct, PKA-independent manner, and there is a cyclic nucleotide-binding domain (CNDB) on the C-terminal of each subunit of the channel [20]. However, intracellular dialysis of cAMP (100 µM) or Rp-cAPM (100 µM), a hydrolysis-resistant cAMP analog, did not occlude the enhancement of *Ih* by SKF83959 (Figs. 5D, 5E), indicating that SKF83959-induced enhancement of *Ih* current is independent of intracellular cAMP-related mechanisms. With cAMP (100 µM) or Rp-cAPM (100 µM) present in the recording pipettes, perfusion with SKF83959 (50 µM) increased the amplitude of *Ih* by 70.7±19.6% (n = 6, paired *t* test, *P*<0.01) or by 55.1±8.3% (n = 6, paired *t* test, *P*<0.001), respectively.

### Effects of SKF83959 on somatic excitability of hippocampal CA1 pyramidal neurons

**Figure 6:**
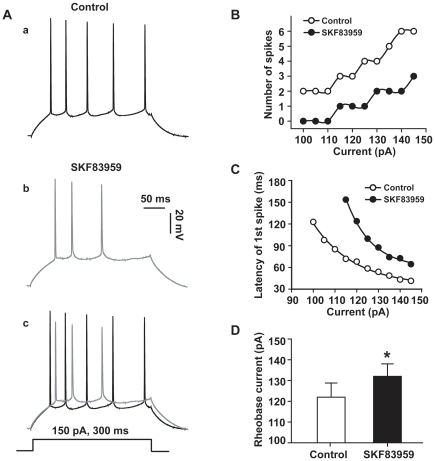
SKF83959 suppressed the somatic excitability of CA1 pyramidal neurons in hippocampal slices. A. Train of action potentials of a representative neuron in response to a prolonged depolarization current pulse (150 pA, 300 ms) prior to (a) and after (b) perfusion with SKF83959 (50 µM). The resting potential was −69 mV in (a). The membrane potential was compensated by injecting steady hyperpolarizing current in (b). The two traces were superimposed at the bottom (c). B. Plot of the number of action potentials against the current intensities in another neuron. C. Plot of the latency of the first spike against the current intensities in the same neuron shown in B. The latency was defined as the time between the onset of depolarizing current pulse and the time of threshold of the first spike. D. Bar graph showing the rheobase currents measured prior to and after perfusion with SKF83959 (50 µM). *P<0.05 vs. Control.

Repetitive discharge of CA1 pyramidal neurons was elicited by injecting prolonged depolarizing current pulses. Comparing the records prior to and after bath application of SKF83959 (50 µM) reveals that the drug markedly reduced the number and the amplitude of action potentials evoked by the depolarizing current pulses (Figs. 6A, 6B), and increased the latency of the first spike firing in the train (Fig. 6C). Furthermore, the rheobase current (the minimum current to evoke a single action potential) was also increased from 122±7 pA to 132±7 pA (n = 5, paired *t* test, *P*<0.05) (Fig. 6D).

**Figure 7:**
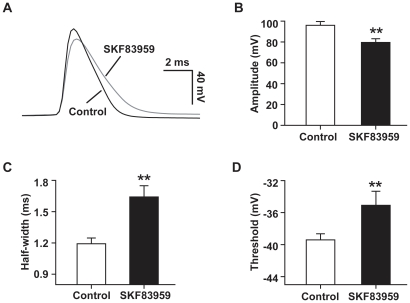
Effect of SKF83959 on action potentials of CA1 pyramidal neurons in hippocampal slices. A. Superimposed action potentials elicited in a representative neuron prior to and after perfusion with SKF83959 (50 µM). For comparison, the membrane potential was compensated. B. Bar graph showing the amplitude of action potentials prior to and after perfusion with SKF83959 (50 µM). The amplitude was defined as the voltage difference between the threshold and peak of the action potential. C. Bar graph showing the half-width of action potentials prior to and after perfusion with SKF83959. The half-width was measured as the width of half-maximal spike amplitude. D. Bar graph showing the threshold of action potentials prior to and after perfusion with SKF83959 (50 µM). The threshold was defined as the first point on the upstroke of action potential with a rising rate exceeded 50 mV/ms. In B, C, D, n = 8, **P<0.01 vs. Control.

To further characterize the influence of SKF83959 on action potential, single spike was elicited by injecting depolarizing current pulse. Perfusion of SKF83959 (50 µM) slowed down both the upstroke and repolarizing phases of the spike. As a result, the action potential was broadened (Fig. 7A, 7C). Pooled data from 8 neurons showed that SKF83959 significantly reduced the amplitudes of action potential (from 96.3±2.1 to 81.8±2.7 mV, paired *t* test, *P*<0.001) and increased the half-width of the spikes from 1.2±0.1 to 1.7±0.1 ms, paired *t* test, *P*<0.01) (Fig. 7C). In addition, the threshold of action potential was also significantly raised from −39.2±0.5 to −34.7±1.2 mV (*P*<0.01, paired *t* test, Fig. 7D). All the above data indicated that the somatic excitability of hippocampal CA1 pyramidal neurons was dramatically reduced by SKF83959.

## Discussion

In the present study we characterized the electrophysiological effects of SKF83959, an atypical D_1_-like receptor agonist, on the passive membrane properties and excitability of hippocampal CA1 pyramidal neurons. The major findings are summarized as following: (1) SKF83959 caused depolarizing response associated with a reduction of input resistance; (2) SKF83959-induced depolarization was mediated mainly by an enhancement of *Ih* via a D_1_-like receptor-independent mechanism. (3) SKF83959 reduced the neuronal excitability.

In the present study we demonstrate that SKF83959 causes a concentration-dependent depolarizing response in rat hippocampal CA1 pyramidal neurons. Furthermore, we found that the effect was sensitive neither to bath application of TTX, SCH23390, raclopride, mesulergine, prazosin, nor to intracellular dialysis of GDP-β-S. Therefore, it is clear that the response of SKF83959 is not mediated by interaction of SKF83959 with G-protein coupled receptors. This implicates that the drug's effect was mediated by an action on ion channels responsible for setting the resting membrane potential, such as TASK-1 channels, HCN channels, etc.[20], [22]. Indeed, in the current-clamp experiments we showed that SKF83959 enhanced the voltage sag caused by prolonged hyperpolarizing current pulse, which is a hallmark of HCN channel activation [20]. In voltage-clamp experiments we demonstrated that SKF83959 enhanced the *Ih* current. Furthermore, the enhancement of *Ih* by SKF83959 is also insensitive to bath application of SCH2339 or intracellular dialysis of GDP-β-S or GppNHp (Figs. 5A, 5B, 5C), suggesting that the effect was independent of activation of D_1_-like receptors. Therefore, both the results form current- and voltage-clamp experiments suggest that SKF83959-induced depolarization mainly due to the enhancement of *Ih* current via DA receptor-independent mechanisms.

There is a cyclic nucleotide-binding domain (CNDB) on the C-terminal of each HCN channel subunit, and the binding of cAMP directly facilitates activation of HCN channels [20]. In fact, the effects of SKF83959 on *Ih* resemble those caused by cAMP: (1) causing a right shift of the activation curve of *Ih* (Fig. 4A), and (2) speeding up the activation kinetics (Fig. 4B). However, intracellular dialysis of high concentrations of cAMP or Rp-cAPM did not occlude the enhancement of *Ih* by SKF83959 (Figs. 5D, 5E), suggesting that the compound's effect was not mediated by intracellular cAMP. At the present, several specific blockers of HCN channels including ZD7288 are commercially available, which have been developed as “heart rate-lowering” agents to block a pacemaker current *I*_f_ in cardiomyocyte [23]. On the other hand, few small molecule compounds other than cyclic nucleotides were found thus far to enhance *Ih* current. The anticonvulsant lamotriqine was reported to preferentially alter the dendritic excitability of hippocampal CA1 pyramidal neurons through increase of *Ih* current (Poolos et al. 2002). To our knowledge, our study provides the first evidence that SKF83959 represents another small molecule activator of *Ih* current. The molecular target of SKF83959 on HCN channel, however, remains to be identified.

Theoretically, a depolarizing response would increase the spontaneous firing and the number of action potentials evoked by depolarizing pulses. In the present study, spontaneous firing was never observed during SKF83959-induced depolarization. In contrast, we found that SKF83959 significantly reduced the number (Figs. 6A, 6B)and amplitude (Figs. 7A, 7B) of action potentials evoked by depolarizing current pulses, prolonged the latency of the first spike (Fig. 6C) and increased the rheobase current (Fig. 6D) as well as the threshold of action potential firing (Fig. 7D). The somatic recordings demonstrate that SKF83959 suppress the excitability of postsynaptic CA1 pyramidal neurons. However, the effects do not seem to be due to the increased *Ih* current. A non-uniform gradient of HCN channel distribution has been demonstrated in hippocampal CA1 pyramidal neuron with the distal dendrites containing a much higher density of HCN channels than that of the soma [24]. As a result, lamotriqine that increased *Ih* current preferentially reduced dendritic excitability, while minimally affecting the somatic excitability of CA1 pyramidal neuron [25]. We recently demonstrated that SKF83959 exerted potent inhibition on voltage-activated Na^+^ current in acutely dissociated hippocampal pyramidal neurons (data not shown), which may explain the reduction of overall excitability of CA1 pyramidal neurons reported here. The broadening of action potential (Fig. 7A, 7C) could be attributed to the blockade of the delayed rectifier K^+^ current by SKF83959 [16], one of the outward currents responsible for the repolarization of action potentials [26].

The inhibition of neuronal excitability by SKF83959 may contribute to its therapeutic benefits in Parkinson's disease (PD). It was found that the spontaneous activity of striatal neurons in 6-OHDA-lesioned PD rats was several folds higher than in control animals [17]–[19]. Intracellular recording conducted in striatal slices also demonstrated that dopamine-denervation increased neuronal excitability [27]. The hyperactivity of striatal neurons in PD would augment the GABAergic control over the output nucleus of basal ganglia, which may associate with some motor symptoms observed in the disease [28]. SKF83959 may reduce the hyperexcitability of striatal neurons in PD, which, in turn, contributes to its therapeutic effects, including the attenuation of the development of dyskinesia. In addition, inhibition of neuronal excitability by SKF83959 may also improve neuronal survival and contribute to its neuroprotective effect. In the context of PD, neuroprotective effect is important for slowing down the progressive loss of dopamine neurons. It is noted that the present data were obtained from hippocampal pyramidal neurons, whereas Parkinson's disease is more relevant to striatal MSN neurons or DA neurons in SNC. However, hippocampal pyramidal neurons are the most widely studied cell in the central nervous system and serve as excellent models for the general neuronal activity seen in other parts of the nervous system. We believe that the effects found in the present study should occur in other brain regions, such as in striatum where *Ih* channel also widely expressed [29]. Regardless, these receptor-independent mechanisms provide a novel insight for the drug's potent neuroprotective action.

## Materials and Methods

### Ethics Statement

All experimental protocols were approved by the Institutional Animal Care and Use Committees of Shanghai Institute of M ateria Medica,Chinese Academy of Sciences (SIMM-AE-2007-0020) and were in compliance with the Guidelines for the Care and Use of Laboratory Animals (National Research Council, People's Republic of China, 1996).

### Electrophysiological recordings from hippocampal pyramidal neurons

Male Sprague-Dawley rats (2–3 weeks of age) were anesthetized with 10% chloral hydrate (400 mg/kg, i.p.) and decapitated. The brain was rapidly removed and placed in an ice-cold ACSF containing the following (in mmol/L): NaCl 119, KCl 2.5, CaCl_2_ 2.5, MgSO_4_ 1.3, NaH_2_PO_4_ 1, NaHCO_3_ 26.2, and glucose 11, bubbled with a gas mixture (95%O_2_ and 5%CO_2_). Transverse hippocampal slices (350 µm) were cut using a M752 vibroslice (Campden Instruments Ltd., UK), and incubated in the ACSF at room temperature. After equilibration for at least 1 hour, one piece of the slices was transferred to recording chamber and perfused with oxygenated ACSF at a rate of 2–3 ml/min.

Whole-cell recordings of CA1 pyramidal neurons were made under a DIC upright microscope (BX51WI, Olympus, Japan) using a MultiClamp 700A amplifier. The recording electrodes (a tip resistance of 3–5 MΩ) were pulled from borosilicate glass pipettes (Sutter Instrument, USA) using a Flaming/Brown micropipette puller (model P-97, Sutter Instrument, USA), and filled with a pipette solution containing (in mmol/L): K-gluconate 140, CaCl_2_ 0.1, MgCl_2_ 2, HEPES 10, ATP·K_2_ 2, GTP·Na_3_ 0.1, and EGTA 1 (pH 7.25 with KOH). Current-clamp recording was performed at 32–34°C as previously described [30]. For recording *Ih* current, the slice was perfused with a modified ACSF containing (in mmol/L): NaCl 110, KCl 5, NaHCO_3_ 24, MgCl_2_ 1, Glucose 10, TEA-Cl 10, AP-4 5, TTX 0.5, CdCl_2_ 1, BaCl_2_ 0.5, which blocked all the other voltage-activated currents. I*h* current was elicited every 30 s with a series of 1.5-s hyperpolarizing voltage steps from a holding potential of −45 mV to −105 mV. Leakage and capacitive currents were subtracted digitally off-line by scaling the trace evoked by voltage steps from −20 to −30 mV. Signals were filtered at 2−10 kHz and sampled at frequencies of 10−40 kHz using pClamp 9.2 software (Molecular Device, Sunnyvale, CA) via a DigiData-1322A interface (Molecular Device, Sunnyvale, CA), and stored in an IBM compatible computer.

### Data acquisition and analysis

Data are presented as mean±S.E.M. Statistical significance was assessed using paired or unpaired Student's *t* test or ANOVA, and *P*<0.05 was considered to be significant. Data analyses were performed using the software Excel 2003 (Microsoft) and Origin 8.0.

### Drug application

(±)-SKF83959, R-(+)-SCH23390 hydrochloride, S(−)-raclopride (+)-tartrate salt, prazosin hydrochloride, mesulergine hydrochloride, GDP-β-S, GppNHp, (−)-bicuculline methiodide and tetrodotoxin were purchased from Sigma-Aldrich China Inc. ZD7288 was purchased from Tocris Bioscience.

For preparing stock solutions, SKF83959 and prazosin were dissolved in dimethylsulfoxide (DMSO), other drugs in distilled water. The solutions were stored at −20°C, and diluted in ACSF to desired concentrations before use. DMSO with a final concentration less than 0.1% had no detectable effect on the membrane properties and *Ih* current of the recorded neurons, nor did the receptor antagonists at desired concentrations. Most drugs were delivered to the slice through perfusion, expect GDP-β-S, GppNHp, cAMP and Rp-cAMP, which were added in the pipette solution, and dialyzed into the neurons recorded.
